# Supplementary material for: Exhaled Breath Analysis in Lymphangioleiomyomatosis by Real-Time Proton Mass Spectrometry
Source: Int J Mol Sci. 2025 Jun 23;26(13):6005. doi: 10.3390/ijms26136005 (PMC12249990; doi:10.3390/ijms26136005)
Supplement: Supplementary file 1 [file ijms-26-06005-s001.zip › ijms-3559025-supplementary.pdf]

Supplementary Materials

Exhaled Breath Analysis in Lymphangioleiomyomatosis by Real-Time Proton Mass Spectrometry

Malika Mustafina [1,2,3,*], Artemiy Silantyev [4], Marina Makarova [2,5], Aleksandr Suvorov [4], Alexander Chernyak [2], Zhanna Naumenko [2], Pavel Pakhomov [6], Ekaterina Pershina [4,7], Olga Suvorova [8], Anna Shmidt [8], Anastasia Gordeeva [8], Maria Vergun [4], Olesya Bahankova [1], Daria Gognieva [1,3,4], Aleksandra Bykova [1,3], Andrey Belevskiy [5], Sergey Avdeev [2,8], Vladimir Betelin [3] and Philipp Kopylov [1,3,4]

1 Department of Cardiology, Functional and Ultrasound Diagnostics, I.M. Sechenov First Moscow State Medical University (Sechenov University), 119991 Moscow, Russia; larkrize1@gmail.com (O.B.); gognieva_d_g@staff.sechenov.ru (D.G.); aabykowa@yandex.ru (A.B.)
2 Pulmonology Research Institute under the Federal Medical and Biological Agency of Russia, 115682 Moscow , Russia; achi2000@mail.ru (A.C.); naumenko_janna@mail.ru (Z.N.);
3 Research Institute for Systemic Analysis of the Russian Academy of Sciences, 117218 Moscow, Russia; betelin@niisi.msk.ru (V.B.)
4 World-Class Research Center "Digital Biodesign and Personalized Healthcare", I.M. Sechenov First Moscow State Medical University (Sechenov University), 119991 Moscow, Russia; artsilan@gmail.com (A.S.); suvorov_a_yu_1@staff.sechenov.ru (A.S.); vergun_m_a@staff.sechenov.ru (M.V.); kopylov_f_yu@staff.sechenov.ru (P.K.)
5 N.I. Pirogov Russian National Research Medical University, 1 Ostrovityanova str., Bldg. 6, 117513 Moscow, Russia; pulmobas@yandex.ru (A.B.); mma123@list.ru (M.M.)
6 National Medical Research Center of Otorhinolaryngology, Federal Medical and Biological Agency of Russia, 123182 Moscow, Russia; pavel.v.pakhomov@gmail.com (P.P.)
7 First Moscow City Hospital named after N.I. Pirogov, 119049 Moscow, Russia; pershina86@mail.ru (E.P.)
8 Pulmonology Department, I.M. Sechenov First Moscow State Medical University (Sechenov University), 119991 Moscow, Russia; olga.a.suvorova@mail.ru (O.S.); a_e_schmidt@mail.ru (A.S.); gordeeva.anast.aleks@gmail.com (A.G.); avdeev_s_n@staff.sechenov.ru (S.A.)
* Correspondence: mustafina_m_kh@staff.sechenov.ru; Tel.: +7-916-4785-942

Table S1. Selected VOCs for functional and clinical endpoints in the forced expiratory maneuver and the normal quiet breathing by the XGBoost algorithm.

End point	m/z	VOC name	m/z error, ppm	Annotation method	Feature importances	
					Forced expiratory maneuver	Normal quiet breathing
LAM diagnosis	44.98976883	NA			0.00677846	0.00683706
	50.00064038	NA			0.00679701	0.00683653
	82.07126739	NA			0.00681889	0.00683042
	90.06287087	Lactic acid	346.3*	HMDB	0.00679180	0.00683309
	99.06710588	NA			0.00679757	0.00682748
	113.1295536	NA			0.00685565	0.00691124
	126.9962244	NA			0.00684542	0.00682354
	150.1015017	NA			0.00683700	0.00685841
	329.8204103	NA			0.00682365	0.00682728
Pneumothorax	44.98976883	NA			0.00704518	0.00741868
	103.0752188	Isopropyl acetate	−1.802563868	[1]	0.00695703	0.00701847
	124.9782102	NA			0.00697791	0.00734019
	133.0957596	2-Ethoxyethyl acetate	73.56372765	[1]	0.00756860	0.00716509
	229.2192372	NA			0.00699943	0.00702694
FEV1/FVC < LLN	50.00064038	NA			0.00693616	0.00696177
	55.03724695	NA			0.00685126	0.00689933
	63.01941085	Dimethyl sulfide	−110.042076	PTR-MS VMS	0.00696004	0.00684350
	77.05561728	NA			0.00681738	0.00685159
	116.1108686	NA			0.00689843	0.00697465
	129.0712551	NA			0.00692103	0.00686908
	134.0767613	NA			0.00682938	0.00687842
	141.1255123	2-Nonenal	−13.30500054	[2]	0.00687696	0.00700937
	163.1152317	NA			0.00681533	0.00690088
	459.1527823	NA			0.00682881	0.00685980
FEV1 < LLN	43.63804260	NA			0.00681570	0.00712937

End point	m/z	VOC name	m/z error, ppm	Annotation method	Feature importances	
					Forced expiratory maneuver	Normal quiet breathing
	48.04336193	O-methylhydroxylamine	−22.4145609	PTR-MS VMS	0.00692541	0.00684413
	173.1514888	Isomers of Oxononanoic acid	197.6660106	[3]	0.00679017	0.00688988
	235.1524095	NA			0.00681568	0.00683073
	377.0803750	NA			0.00685988	0.00685590
	445.1010852	NA			0.00683779	0.00685852
RV/TLC > LLN	74.04986341	N-methylacetamide	−138.0699801	PTR-MS VMS	0.00855004	0.02698160
	139.0780609	NA			0.00295458	0.01525660
RV > LLN	42.02864698	Acetonitrile	−124.3556562	[4]	0.00290517	0.00849818
	70.07278854	NA			0.00949611	0.00048547
	86.10863088	NA			0.00298550	0.01013470
	159.0671816	NA			0.01527170	0.00051611
	235.1524095	NA			0.00210955	0.00139279
	285.2644931	NA			0.00954684	0.00710494
DLCO < LLN	43.63804260	NA			0.00680456	0.00689998
	48.04336193	O-methylhydroxylamine	−22.4145609	PTR-MS VMS	0.00687678	0.00690884
	60.05278301	NA			0.00682123	0.00691833
	68.06002575	NA			0.00681663	0.00696516
	76.04698747	NA			0.00696591	0.00707272
	85.09458123	NA			0.00691375	0.00691726
	179.1428730	NA			0.00691423	0.00700770
	225.0532236	NA			0.00689601	0.00685901
	377.0803750	NA			0.00687714	0.00687393
Volume of lung cysts**	49.00395161	Methanethiol	−137.6176121	PTR-MS VMS	0.00705556	0.00701770
	56.04938580	NA			0.00696473	0.00714787
	111.1054554	NA			0.00698137	0.00721914
	123.0373884	NA			0.00696063	0.00707595
	129.0712551	NA			0.00695566	0.00699561
	207.1795575	NA			0.00699336	0.00719078
	257.2648823	NA			0.00694597	0.00704281
	429.0782954	NA			0.00695728	0.00697739
	459.1527823	NA			0.00694124	0.00699999
	533.1373222	NA			0.00725726	0.00734667
B1 and B10 dist. average lumen area***	42.58494405	NA			0.00736113	0.00753056
	49.00395161	Methanethiol	−137.6176121	PTR-MS VMS	0.00698881	0.00747585
	57.06132738	Isobutene	−150.655708	PTR-MS VMS	0.00722638	0.00729180
	72.06134628	NA			0.00698148	0.00705474
	73.06335251	Methyl ethyl ketone	−20.35726078	[5]	0.00720196	0.00705437
	82.07126739	NA			0.00696445	0.00700000
	91.05764653	2-Butanethiol	−34.75095306	PTR-MS VMS	0.00699667	0.00724412
	181.0034270	Oxidized Lipid Fragment	−198.72	HMDB	0.00699667	0.00699890
	297.0584547	NA			0.00698894	0.00708142
	356.0707054	NA			0.00715780	0.00702684
	431.0861313	NA			0.00696899	0.00776823

*Mass error is outside the range but close to 200 ppm; ** Volume of cysts in both lungs; *** Average lumen area of the distal part of the B1 and B10 bronchi of the right lung. DLCO: diffusing capacity of the lungs for carbon monoxide; FEV_1: forced expiratory volume in 1 second; FVC: forced vital capacity; HMDB: Human Metabolome Database; NA: not available; LLN: lower limit of normal (z-score < −1.645); PTR-MS VMS: proton mass-spectrometry view mass calculator; RV: residual volume; TLC: total lung capacity.

Table S2. Performance metrics for normal and forced breathing.

	Performance Metrics for Normal breathing		Performance Metrics for Forced breathing	
**Pneumothorax**	Max Error	2.0001	Max Error	2.0001
	RMSE	0.4000	RMSE	0.4000
	Explained variance	0.9608	Explained variance	0.9608
	R^2	0.9608	R^2	0.9608
**FEV1/FVC < LLN**	Max Error	0.6233	Max Error	0.6232
	RMSE	0.0786	RMSE	0.0786
	Explained variance	0.9981	Explained variance	0.9981
	R^2	0.9981	R^2	0.9981
**FEV1 < LLN**	Max Error	1.8100	Max Error	1.8100
	RMSE	0.2281	RMSE	0.2281
	Explained variance	0.9840	Explained variance	0.9840
	R^2	0.9840	R^2	0.9840
**RV/TLC > LLN**	Max Error	1.5267	Max Error	1.5265
	RMSE	0.2053	RMSE	0.2053
	Explained variance	0.9793	Explained variance	0.9793
	R^2	0.9793	R^2	0.9793
**RV > LLN**	Max Error	0.3033	Max Error	0.3034
	RMSE	0.0411	RMSE	0.2053
	Explained variance	0.9988	Explained variance	0.0411
	R^2	0.9988	R^2	0.9988
**DLCO < LLN**	Max Error	3.7799	Max Error	3.7800
	RMSE	0.9737	RMSE	0.4785
	Explained variance	0.9793	Explained variance	0.9737
	R^2	0.9737	R^2	0.9737
**Volume of lung cysts***	Max Error	0.0027	Max Error	0.0022
	RMSE	0.0009	RMSE	0.0009
	Explained variance	1.0000	Explained variance	1.0000
	R^2	1.0000	R^2	1.0000
**B1 and B10 dist. average lumen area****	Max Error	0.0024	Max Error	0.0015
	RMSE	0.0007	RMSE	0.0005
	Explained variance	1.0000	Explained variance	1.0000
	R^2	1.0000	R^2	1.0000

* Volume of cysts in both lungs; ** Average lumen area of the distal part of the B1 and B10 bronchi of the right lung. DLCO: diffusing capacity of the lungs for carbon monoxide; FEV_1: forced expiratory volume in 1 second; FVC: forced vital capacity; HMDB: Human Metabolome Database; NA: not available; LLN: lower limit of normal (z-score < −1.645); PTR-MS VMS: proton mass-spectrometry view mass calculator; RV: residual volume; R^2—Coefficient of Determination; RMSE—Root Mean Squared Error; TLC: total lung capacity

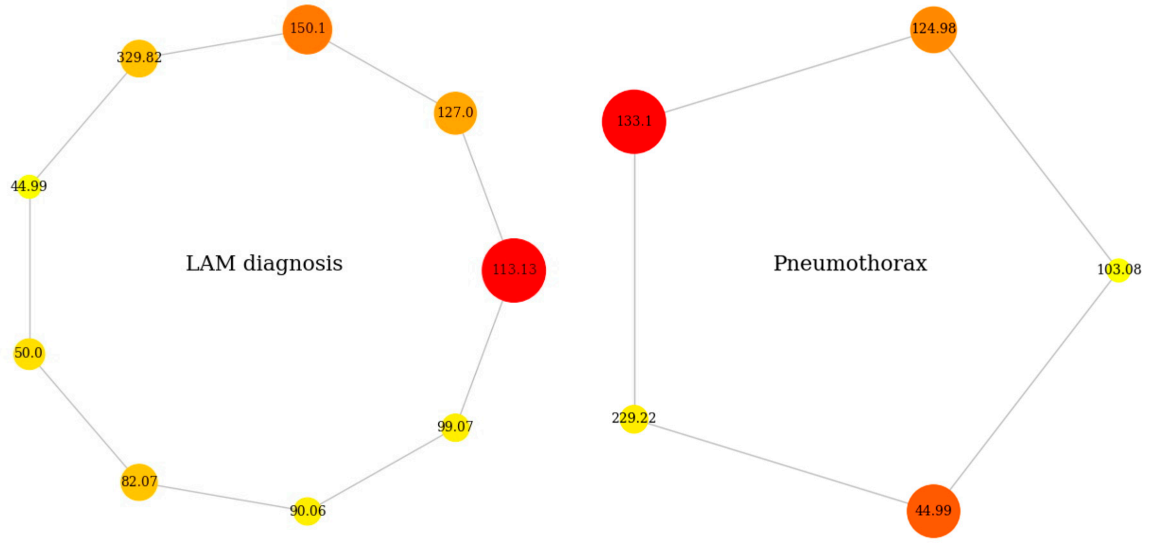

(**A**) (**B**)

Figure S1. Summarized importance of features for LAM diagnosis and pneumothorax outcome in LAM. (**A**) LAM diagnosis. (**B**) Pneumothorax. The size of the node is determined by the total importance of the feature, both during a forced and normal expiratory maneuver (the higher the importance of the feature, the larger the size of the node). The color of a node is determined by the frequency of occurrence of the feature in different outcomes. Bright red nodes reflect the frequency of the feature in more than 2 outcomes. The number in the node indicates the mass per charge (m/z) of the volatile organic compound (VOC).

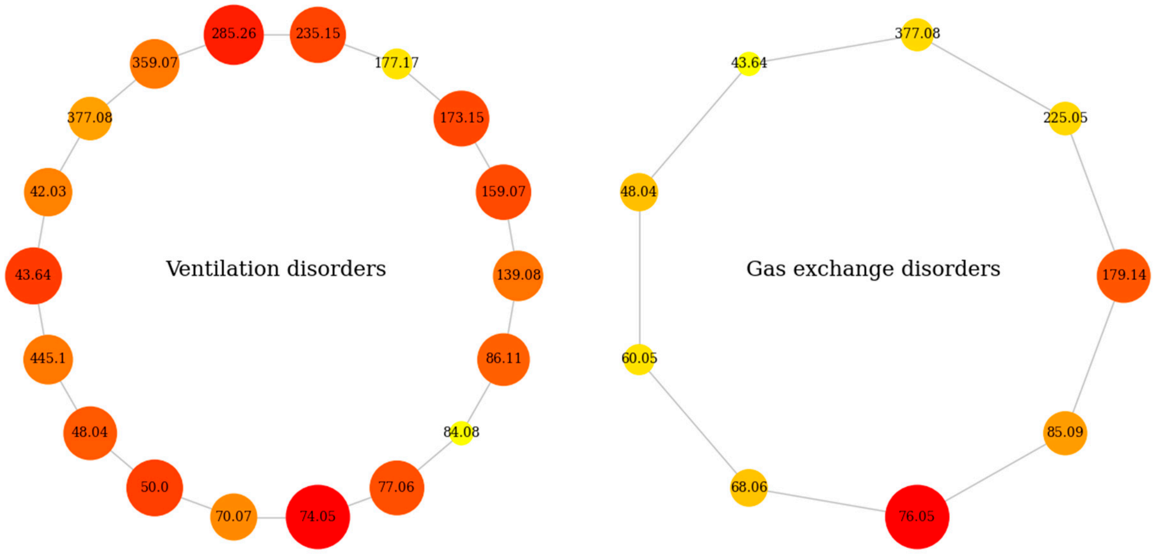

(**A**) (**B**)

Figure S2. Summarized importance of features for respiratory outcomes in LAM. (**A**) Ventilation disorders—obstructive disorders (FEV1 /FVC < LLN) and air trapping (RV/TLC > LLN and RV > LLN). (**B**) Gas exchange dysfunction (DLCO < LLN). DLCO: diffusing capacity of the lungs for carbon monoxide; FEV1: forced expiratory volume in 1 second; FVC: forced vital capacity; LLN: lower limit of normal (z-score < −1.645); RV: residual volume; TLC: total lung capacity. The size of the node is determined by the total importance of the feature, both during a forced and normal expiratory maneuver (the higher the importance of the feature, the larger the size of the node). The color of a node is determined by the frequency of occurrence of the feature in different outcomes. Bright red nodes reflect the frequency of the feature in more than 2 outcomes. The number in the node indicates the mass per charge (m/z) of the volatile organic compound (VOC).

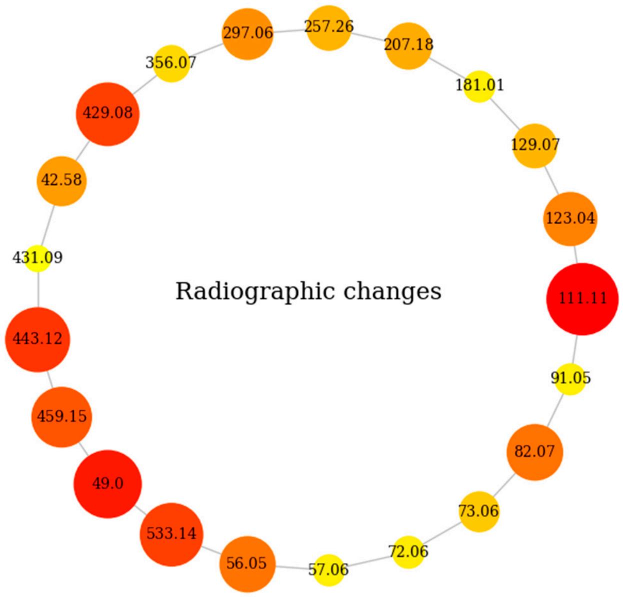

Figure S3. Summarized importance of features for radiographic changes in LAM Radiographic changes—Volume of cysts in both lungs and the average lumen area of the distal part of the B1 and B10 bronchi of the right lung. D. The size of the node is determined by the total importance of the feature, both during a forced and normal expiratory maneuver (the higher the importance of the feature, the larger the size of the node). The color of a node is determined by the frequency of occurrence of the feature in different outcomes. Bright red nodes reflect the frequency of the feature in more than 2 outcomes. The number in the node indicates the mass per charge (m/z) of the volatile organic compound (VOC).

References

1. van Mastrigt, E.; Reyes-Reyes, A.; Brand, K.; Bhattacharya, N.; Urbach, H.P.; Stubbs, A.P.; de Jongste, J.C.; Pijnenburg, M.W. Exhaled breath profiling using broadband quantum cascade laser-based spectroscopy in healthy children and children with asthma and cystic fibrosis. *J. Breath Res.* **2016**, *10*, 026003. https://doi.org/10.1088/1752-7155/10/2/026003.
2. van Berkel, J.J.B.N. There's Something in the Air: Volatile Organic Compounds in Exhaled Breath in Pulmonary Diseases. Doctoral Thesis, Maastricht University, Maastricht, 2010. https://doi.org/10.26481/dis.20101103jb
3. Weber, R.; Perkins, N.; Bruderer, T.; Micic, S.; Moeller, A. Identification of Exhaled Metabolites in Children with Cystic Fibrosis. *Metabolites* **2022**, *12*, 980. https://doi.org/10.3390/metabo12100980.
4. Natal Jorge, R.M.; Tavares, J.M.R.; Barbosa, M.P.; Slade, A.P. (Eds.). *Technology and Medical Sciences*, 1st ed.; Taylor & Francis: London, United Kingdom, 2011. https://doi.org/10.1201/b11330.
5. Kos, R.; Brinkman, P.; Neerincx, A.H.; Paff, T.; Gerritsen, M.G.; Lammers, A.; Kraneveld, A.D.; Heijerman, H.G.M.; Janssens, H.M.; Davies, J.C.; et al. Amsterdam Mucociliary Clearance Disease (AMCD) Research Group and the Amsterdam UMC Breath Research Group. Targeted exhaled breath analysis for detection of *Pseudomonas aeruginosa* in cystic fibrosis patients. *J. Cyst. Fibros.* **2022**, *21*, e28–e34.
